# The evolution of sexually transmitted infections in Romania

**DOI:** 10.1186/1471-2334-13-S1-O36

**Published:** 2013-12-16

**Authors:** Vasile Benea, Simona Roxana Georgescu, Viorica Gheorghiu, Mircea Tampa, Mihaela-Anca Benea-Mălin

**Affiliations:** 1 Department of Dermatology, Clinical Hospital of Infectious and Tropical Diseases “Dr. Victor Babeş”, Bucharest, Romania; 2 Public Health Institute Bucharest, Romania

## Background

The objective of the present study was to analyze the evolution of incidence of some sexually transmitted infections (STI) in Romania in the transition period.

## Methods

We had in view to evaluate the evolution of incidence of syphilis, gonorrhea, *C trachomatis* genitally infections and HIV infection/AIDS and to identify the main factors implicated in this evolution.

## Results

In 2012 were recorded 1,702 new cases of syphilis. The incidence of syphilis has risen steadily from 7.1 per ten thousand in 1986 to 19.8 in 1989 and to 58.5 in 2002 and decreased to 7.93 per ten thousand in 2012. The incidence of congenital syphilis was also increasing, from no cases in 1986 to 423 cases in 2001 and (after introduction of new criteria in 2004) decreased to 10 cases in 2011. Paradoxically, the incidence of gonorrhea is decreasing, from 57.4 per ten thousand in 1986 to 35.7 in 1989 and to 1.49 per ten thousand in 2012 (314 cases). In 2012 57 new cases of *C trachomatis* genitally infections were reported (0.62 per ten thousand). The prevalence of HIV infection in patients with STI tested at Dermato-venerology Centre Bucharest is around 0.51% (3.22% in 2012).

Those at greatest risk for STD are the young, economically deprived, residents of the inner city.

## Conclusion

STI are a public health problem of major significance in Romania. Between mains factors that promote the increasing of STI (the incidence of gonorrhea and *C trachomatis* genitally infections is underestimated due to the unreference of all cases) are the modification of sexual behavior, prostitution, degradation of socioeconomic condition, and deficiencies in health behavior.

